# Knowledge, Attitudes, and Practices Regarding COVID-19 among Healthcare Workers in Uganda: A Cross-Sectional Survey

**DOI:** 10.3390/ijerph18137004

**Published:** 2021-06-30

**Authors:** Onesmus Kamacooko, Jonathan Kitonsa, Ubaldo M. Bahemuka, Freddie M. Kibengo, Anne Wajja, Vincent Basajja, Alfred Lumala, Ayoub Kakande, Paddy Kafeero, Edward Ssemwanga, Robert Asaba, Joseph Mugisha, Benjamin F. Pierce, Robin J. Shattock, Pontiano Kaleebu, Eugene Ruzagira

**Affiliations:** 1 Medical Research Council/Uganda Virus Research Institute and London School of Hygiene and Tropical Medicine Uganda Research Unit, Entebbe P.O. Box 49, Uganda; Jonathan.Kitonsa@mrcuganda.org (J.K.); Ubaldo.Bahemuka@mrcuganda.org (U.M.B.); Freddie.Kibengo@mrcuganda.org (F.M.K.); Anne.Wajja@mrcuganda.org (A.W.); Vincent.Basajja@mrcuganda.org (V.B.); Ayoub.Kakande@mrcuganda.org (A.K.); Paddy.Kafeero@mrcuganda.org (P.K.); Joseph.Mugisha@mrcuganda.org (J.M.); Pontiano.Kaleebu@mrcuganda.org (P.K.); Eugene.Ruzagira@mrcuganda.org (E.R.); 2 Kitovu Hospital, Masaka P.O. Box 524, Uganda; lumalaalfred@gmail.com; 3 Villa Maria Hospital, Masaka P.O. Box 32, Uganda; essemwang@gmail.com; 4 Our Lady of Consolata Kisubi Hospital, Entebbe P.O. Box 40, Uganda; asabdoc@gmail.com; 5 Department of Infectious Disease, Imperial College London, Norfolk Place, London W2 1PG, UK; b.pierce@imperial.ac.uk (B.F.P.); r.shattock@imperial.ac.uk (R.J.S.)

**Keywords:** COVID-19, SARS-COV-2, healthcare workers, knowledge, attitude, practices, Uganda

## Abstract

Healthcare workers (HCWs) are at high risk of COVID-19. However, data on HCWs’ knowledge, attitudes, and practices (KAP) toward COVID-19 are limited. Between September and November 2020, we conducted a questionnaire-based COVID-19 KAP survey among HCWs at three hospitals in Uganda. We used Bloom’s cut-off of ≥80% to determine sufficient knowledge, good attitude, and good practice, and multivariate Poisson regression with robust variance for statistical analysis. Of 717 HCWs invited to participate, 657 (91.6%) agreed and were enrolled. The mean age (standard deviation) of enrollees was 33.2 (10.2) years; most were clinical HCWs (64.7%) and had advanced secondary school/other higher-level education (57.8%). Overall, 83.9% had sufficient knowledge, 78.4% had a positive attitude, and 37.0% had good practices toward COVID-19. Factors associated with KAP were: Knowledge: being a clinical HCW (aRR: 1.12; 95% CI: 1.02–1.23) and previous participation in health research (aRR: 1.10; 95% CI: 1.04–1.17); Attitude: age > 35 years (aRR: 0.88; 95% CI: 0.79–0.98); Practice: being a clinical HCW (aRR: 1.91; 95% CI: 1.41–2.59). HCWs in Uganda have good knowledge and positive attitude but poor practices towards COVID-19. Differences in COVID-19 KAP between clinical and non-clinical HCWs could affect uptake of COVID-19 interventions including vaccination.

## 1. Introduction

Coronavirus disease 2019 (COVID-19) caused by the SARS-CoV-2 novel human coronavirus was first reported in December 2019 in Wuhan, China [1,2]. The disease quickly spread to all continents and on 11 March 2020 was declared a global pandemic by the World Health Organization (WHO) [3]. As of 1 May 2021, 150,110,310 confirmed cases of COVID-19, including 3,158,792 deaths, had been reported to the WHO globally [4]. Of these, over 4.5 million confirmed cases including more than 121,000 deaths were in sub-Saharan Africa. Although initially slow to spread in Africa, confirmed cases of COVID-19 on the continent are rising steadily [5].

The rapid spread of SARS-CoV-2 may be attributed to its high transmissibility, asymptomatic virus shedding, high numbers of patients with mild symptoms, and super-spreading events [6,7]. There is currently no effective drug approved for the treatment of COVID-19 [7,8]. Therapy consists of supportive care while several drugs are being evaluated in clinical trials [9]. Preventive measures include social distancing, practicing hand hygiene and respiratory etiquette, wearing a facemask in public settings, and monitoring and self-isolation for people suspected to have infection [10]. Several COVID-19 vaccine candidates have demonstrated very good efficacy in randomized clinical trials [11,12,13,14,15,16,17]. At least seven different vaccines have been rolled out in several countries with over 13 million doses administered in Africa to date [18,19]. Despite this progress, there are concerns that access to COVID-19 vaccines in Africa will be slow due to higher-income countries pre-emptively buying up vaccine supplies, low vaccine-manufacturing capacity, and barriers to widespread delivery and uptake of the vaccines across the continent [20,21]. Given this prospect, African countries will likely continue to rely on the implementation of existing preventive measures to contain the spread of SARS-CoV-2 for the foreseeable future. Additionally, vaccines may not directly stop transmission of the virus, and therefore, social behavioral measures will remain necessary in the control of the pandemic [22].

Healthcare workers (HCWs) are at the forefront in the containment of COVID-19 and hence are at increased risk of exposure to SARS-CoV-2. However, HCWs can also be the source of SARS-CoV-2 to their patients, family, and community members [23]. SARS-CoV-2 infections in HCWs can have a devastating impact, particularly if pathogens are introduced into settings with high numbers of vulnerable individuals, e.g., those with comorbidities [24]. The impact is likely to be particularly big in Africa where healthcare systems are not sufficiently robust to effectively deal with the pandemic [25]. A Global Fund survey of health facilities in 24 African countries including Uganda, found that 50% of the facilities recorded COVID-19 infections among all categories of their staff between April and September 2020 [26]. In the same period, 67% of health facilities reported that up to 10% of staff were absent. Sickness due to COVID-19 or having to quarantine because of exposure to the virus was the primary reason for staff absences in 19% of the facilities. Moreover, only 38% of the surveyed health facilities had the four basic personal protective equipment items, i.e., face masks, disinfectant, gloves and hand sanitizer.

As part of the efforts to mitigate the impact of COVID-19 on healthcare in Africa, it is important that HCWs adhere to the recommended COVID-19 prevention measures. It should be noted, however, that HCWs’ adherence to these measures is mostly influenced by knowledge, attitudes, and practices (KAP) [23,27]. Thus, it is critical that HCWs are equipped with adequate knowledge of COVID-19 prevention policies in order for them to have positive attitudes and appropriate practices that contribute to decreasing risk of infection [28]. Assessing KAP towards COVID-19 among HCWs can aid pandemic control efforts by identifying critical gaps that should be the focus of training policies. A few studies have assessed KAP towards COVID-19 among HCWs in Africa. Whereas some of these studies have reported adequate COVID-19-related KAP among HCWs [29,30], others have found significant gaps [31,32]. In the current study, we assessed KAP towards COVID-19 among HCWs in Uganda with the aim of informing national COVID-19 prevention and control efforts.

## 2. Methods

### 2.1. Study Setting and Design

The study was a cross-sectional survey conducted between September and November 2020. The study was conducted by the Medical Research Council/Uganda Virus Research Institute and London School of Hygiene and Tropical Medicine (MRC/UVRI and LSHTM) Uganda Research Unit in collaboration with three Private-Not-for-Profit (PNFP) community hospitals: (i) Kitovu Hospital, a 248-bed hospital located in Masaka city, about 140 km from Kampala, Uganda’s capital; (ii) Villa Maria Hospital, a 100-bed rural hospital located in Villa Maria, Kalungu District, about 15 km from Masaka city; (iii) Our Lady of Consolata Kisubi Hospital, a 110-bed peri-urban hospital located in Kisubi, Wakiso district, about 28 km from Kampala. At the time of the study, these hospitals had no experience of managing patients with COVID-19.

### 2.2. Study Participants

Study participation was open to all HCWs at the three hospitals including professionals providing direct clinical care to patients, hereby referred to as clinical HCWs (medical doctors, nurses, nursing assistants, allied health professionals, medical/nursing students, and other clinical roles) and all other staff involved in providing non-clinical services, hereby referred to as non-clinical HCWs (administrators, cleaning staff, janitors and other non-clinical roles), who were ≥18 years of age, willing and able to provide informed consent, willing to complete interviewer-administered electronic questionnaires, and not confirmed or suspected to have COVID-19.

### 2.3. Data Collection

Data were collected using a predesigned standardized electronic questionnaire adapted from a previous COVID-19 KAP survey [33] and standard WHO and Uganda Ministry of Health guidance on prevention of COVID-19. The questionnaire was pre-tested among 12 volunteers, and minor changes were made before it was finalized and deployed for the main survey. After obtaining informed consent, trained research assistants administered the questionnaire using encrypted tablets. Questions included items on socio-demographic characteristics, and knowledge, attitudes, and practices towards COVID-19.

### 2.4. Study Variables and Scoring

Socio-demographic characteristics included age, sex, marital status, occupation, role/position at the hospital, highest level of education, household size, and source of information on COVID-19.

Knowledge about COVID-19 was assessed using 9 questions on symptoms, transmission, prevention, and control of COVID-19. The first question allowed for multiple responses where we asked for the most common symptoms of COVID-19 with a listed option assigned a score of 0 if not mentioned and 1 if mentioned. For this assessment, participants were scored on the three main symptoms of fever, cough, and tiredness. Hence, the cumulative score ranged from 0 to 3 points for each participant. Each of the remaining 8 questions was assigned a score of 0 if the response was incorrect or ‘don’t know’, or 1 if the response was correct. Hence, the cumulative score for all 9 questions ranged from 0 to 11 points for each participant. Participants’ overall knowledge was categorized, using Bloom’s cut-off point, as good, if the score was ≥80% (≥8.8 points) [34].

Attitudes towards COVID-19 were assessed using 7 Likert-item questions. The responses were ‘agree’, ‘not sure’, and ‘disagree’ each weighing 2, 1, 0 points, respectively. The cumulative score for all 7 questions ranged from 0 to 14 points for each participant. Overall attitude level was categorized, using Bloom’s cut-off point, as positive, if the score was ≥80% (≥11.2 points) [34].

Practices were assessed using 6 Likert-item questions on the frequency of (i) joining gatherings with people other than household members, (ii) washing hands with soap and water or cleaning them with hand sanitizer, (iii) refraining from shaking hands, (iv) wearing a face mask when at work, (v) washing hands with soap and water or cleaning them with hand sanitizer before and after handling each patient, (vi) avoiding patients with signs and symptoms suggestive of COVID-19. The responses were: twice or more every day, once a day, not every day, and never, each weighing 0, 1, 2, and 3 points, respectively for question (i) and 3, 2, 1, and 0 points, respectively, for question (ii); always, occasionally, and never, each weighing 2, 1, and 0 points, respectively, for questions (iii)–(v) and 0, 1, 2 points, respectively for question (vi). The cumulative score for all 6 questions ranged from 0 to 14 points for each participant. Overall practice level was similarly categorized using Bloom’s cut-off point of ≥80% (≥11.2 points) to determine good practice.

### 2.5. Statistical Analysis

Data from computer tablets were uploaded onto and managed in a RedCap database and subsequently exported to Stata 15.0 (Stata-Corp College Station, TX, USA) for analysis. Participants’ characteristics were summarized descriptively using means, standard deviations (SD) or percentages, as appropriate. Poisson regression with robust variance was used to identify factors associated with knowledge, attitude and practice regarding COVID-19 [35]. Unadjusted (univariable) analyses were conducted, and a likelihood ratio test (LRT) was used to screen for variables to be included in the adjusted (multivariable) model. Only factors for which the associations attained statistical significance at the 15% level in the unadjusted analyses were considered for the multivariable model [36]. Age and sex were included in the multivariable model as a priori confounders. Spearman’s rank correlation coefficient was used to measure the relationship between knowledge, attitude and practice.

## 3. Results

### 3.1. Participant Characteristics

Of the 717 HCWs in the three hospitals, 657 (91.6%) participated in the survey. The mean age of participants was 33.2 (SD, 10.2) years. Most were female 411 (62.6%), married (55.6%), clinical HCWs (64.7%), had advanced secondary/other higher-level education (57.8%), and had no underlying health conditions (85.8%). The most common sources of information on COVID-19 were traditional news media, e.g., radio and television (96%) and social media platforms, e.g., WhatsApp messenger (70.2%). Participants’ sociodemographic characteristics are summarized in Table 1.

### 3.2. Knowledge

Detailed results of knowledge assessment are provided. The most known COVID-19 symptoms were fever (95.0%), cough (88.4%), and shortness of breath (64.1%) while loss of smell (4.1%) and gastro-intestinal symptoms (vomiting, 4.9%, loss of appetite, 7.6%, and diarrhea, 7.6%) were the least known. The majority of the respondents knew that there was no effective cure (97.1%) or vaccine (92.1%) for COVID-19 at the time of the survey; that not all persons with COVID-19 develop severe disease (83.0%), and that persons with COVID-19 can transmit SARS-CoV-2 even if they do not have a fever (91.9%) (Table 2). The average knowledge score was 9.56 (SD, ±1.15). In total, 84.5% of the participants scored ≥80% and were categorized as having sufficient knowledge.

### 3.3. Attitude

Most participants agreed that COVID-19 will be successfully controlled (81.4%); Africans should participate in studies evaluating COVID-19 vaccines (96.4%), and that HCWs should be prioritized for vaccination if a safe and effective COVID-19 vaccine is found (95.6%) (Table 3). The average attitude score was 12.5 (SD, ±1.69). Overall, 72% of the participants scored ≥80% and were categorized as having a positive attitude.

### 3.4. Practice

All the participants reported washing their hands at least twice every day in the two weeks prior to the interview. In the same period, most participants had refrained from shaking hands (85.7%) and worn a mask while at work (90.5%), but only 41.4% had not joined a gathering with non-household members. Among participants whose work involved direct contact with patients, 95.9% reported cleaning their hands before and after handling each patient. However, 42.4% avoided patients with signs and symptoms suggestive of COVID-19 (Table 4). The average practice score of the participants was 10.7 (SD, ±1.85). Overall, only 37.0% of the participants had good practices.

### 3.5. Relationship between Knowledge, Attitude and Practice

There were weak but significant positive correlations between knowledge and attitude (r_s_ = 0.137, *p* =< 0.001), knowledge and practice (r_s_ = 0.116, *p* = 0.003), and attitude and practice (r_s_ = 0.133, *p* = 0.001).

### 3.6. Factors Associated with Knowledge, Attitude and Practice toward COVID-19

We further assessed the factors associated with knowledge, attitude and practice towards COVID-19 among HCWs in Uganda. The results show that Clinical HCWs were more likely to have sufficient knowledge compared to non-clinical HCWs (aRR: 1.12; 95%CI: 1.02–1.23), HCWs who reported having previously participated in health research were more likely to have sufficient knowledge compared to those who did not (aRR: 1.10; 95% CI: 1.04–1.17). In terms of attitude, older participants (>35 years) were less likely to have a positive attitude towards COVID-19 compared to those aged 18–25 years (aRR = 0.88, 95% CI: 0.79–0.98). With regard to practices, Clinical HCWs were more likely to have good practices towards COVID-19 compared to non-clinical HCWs (aRR = 1.91, 95% CI: 1.41–2.59; Table 5).

## 4. Discussion

In this study among HCWs in three PNFP community hospitals in Uganda, we found that 83.9% of the participants had sufficient knowledge of COVID-19. This is higher than the 69.0% observed in a previous KAP survey among HCWs at four University teaching hospitals in Kampala, Uganda [33]. The latter study was conducted relatively early in the pandemic (April 2020) when Uganda had fewer than 100 confirmed cases; hence the observed difference may be attributed to the limited information on COVID-19 at the time. Consistent with the KAP survey among HCWs in University teaching hospitals [33], the main sources of information were traditional news media (TVs, radios, etc.) and social media (Facebook, WhatsApp, etc.).

Consistent with previous studies [30,37], we found that clinical HCWs were more likely to have sufficient knowledge on COVID-19 compared to their non-clinical counterparts. This finding is not surprising given clinical HCWs’ unique training, experience, and knowledge of other infectious diseases. As discussed below, knowledge on COVID-19 is closely related to COVID-19 prevention practices. Since non-clinical HCWs may have direct contact with patients and other HCWs, gaps in knowledge in this population could increase the risk of nosocomial and HCW-to-HCW transmission of SARS-CoV-2 [37,38]. Hence it is critical that COVID-19-related knowledge gaps among non-clinical HCWs are addressed urgently. Participation in health research increases knowledge on general health [39]. This probably explains the finding that previous participation in health research was associated with having sufficient knowledge on COVID-19.

We found that 78.4% of our study participants had a positive attitude towards COVID-19. This is much higher than the 21% previously reported in among HCWs in Uganda [33] and may indicate improvement in attitudes as HCWs learn more about COVID-19. We, however, found that older participants (>35 years) were less likely to have a positive attitude towards COVID-19 compared to those in the youngest age group (18–25 years). This finding is consistent with the results of a study in Nigeria [23] but not others in which either attitude improved with increasing age [40,41,42] or was not impacted by age [33,43]. These contradictory findings may be due to unique contextual factors that require further investigation.

Contrary to findings in other HCW KAP studies [30,33,43,44], and despite the high overall knowledge and attitude scores observed in this study, participants in this study had poor COVID-19 prevention practices. Although most participants practiced good hand hygiene and wore face masks while at work, in the 2 weeks preceding the survey, 58.6% still joined gatherings with non-household members, a practice that significantly increases risk of contracting and spreading SARS-CoV-2. Furthermore, among participants whose work involved direct patient contact, 42.4% avoided patients with signs and symptoms suggestive of COVID-19. These findings may be attributed to lack of personal protective gear [45,46] and knowledge gaps particularly among non-clinical HCWs. Poor COVID-19 prevention practices were more prevalent among non-clinical HCWs compared to clinical HCWs. As noted above, being a non-clinical HCW was associated with having insufficient knowledge on COVID-19.

Our findings show that there were positive correlations between knowledge and attitude, knowledge and practices, and attitude and practices. In the context of COVID-19, knowledge of the disease may influence attitudes and practices, and poor attitudes and practices directly increase the risk of infection with SARS-CoV-2 [47].

## 5. Limitations and Strengths of the Study

A key limitation of our study is that the results may be susceptible to bias due to self-report. Study participants may have provided socially desirable responses especially because data was collected through face-to-face interviews. A further limitation may be that study participants were HCWs in community PNFP hospitals and that, therefore, the findings may not be generalizable to HCWs employed in non-PNFP facilities in Uganda. It is worth noting, however, that the PNFP health sector employs one-third of Uganda’s HCWs [48]. Moreover, we have no reason to believe that HWCs in PNFP facilities differ significantly from their colleagues in non-PNFP settings with regard to education, experience, and other factors that may influence KAPs towards COVID-19. The main strengths of this study were the relatively large sample size which ensured good precision around measured parameters, and the high response rate (92%) and inclusion of HCWs in rural and urban settings which improve the generalizability of the findings.

## 6. Conclusions

Our findings show that HCWs who are employed in community PNFP hospitals in Uganda generally have good knowledge and positive attitudes but poor practices towards COVID-19. To promote uptake of and adherence to COVID-19 prevention measures within hospital and community settings in Uganda, COVID-19 prevention and control programs that target all HCWs should be instituted. These programs should pay special attention to non-clinical HCWs who are likely to be least knowledgeable on the prevention and control of infectious diseases and thus most vulnerable. In particular, the differences observed in this study between clinical and non-clinical HCWs regarding COVID-19 knowledge and practices could result in differential uptake of COVID-19 vaccination in in this population. Hence, it will be critical to closely monitor COVID-19 vaccination programs among HCWs in Uganda and address any barriers to vaccination uptake in a timely manner.

## Figures and Tables

**Table 1:** Participant socio-demographic characteristics.

Characteristic (N = 657)	N (col %)
**Mean age (SD)**	33.2 (10.2)
**Site (Hospital)**	
Kisubi	239 (36.4)
Kitovu	232 (35.3)
Villa-Maria	186 (28.3)
**Sex**	
Male	246 (37.4)
Female	411 (62.6)
**Age group**	
18–25 years	160 (24.4)
26–35 years	290 (44.1)
>35 years	207 (31.5)
**Marital Status**	
Not married	292 (44.4)
Married	365 (55.6)
**Occupation of the respondents**	
Non-clinical HCW	232 (35.3)
Clinical HCW	425 (64.7)
**Education level**	
Ordinary secondary school and below	131 (19.9)
Advanced secondary school/other higher-level	380 (57.8)
University	146 (22.2)
**Family size**	
1–2 persons	255 (38.8)
3–5 persons	255 (38.8)
>5 persons	147 (22.4)
**What are your sources of information on COVID-19 ^¥^**	
Official international health organization websites	87 (13.2)
Official government websites and media	118 (18.0)
News media, e.g., TVs, radios	631 (96.0)
Social media, e.g., WhatsApp	461 (70.2)
Medical journals	101 (15.4)
Other	344 (52.4)
**Previously volunteered to participate in health research**	
No	135 (20.6)
Yes	522 (79.5)
**Has an underlying health condition**	
Yes	93 (14.2)
No	564 (85.8)

N = Number; col = column; % = percentage; ^¥^ Multiple response question.

**Table 2:** Descriptive summary of COVID-19 knowledge among HCWs in Uganda.

Knowledge Questions (N = 657)	Responses		
	True	False	I Don’t Know
	N (%)	N (%)	N (%)
**What are the common symptoms of COVID-19? (Multiple responses allowed)**			
Fever	624 (95.0)	-	-
Cough	581 (88.4)	-	-
Tiredness	214 (32.6)	-	-
Muscle/joint pain	96 (14.6)	-	-
Shortness of breath	421 (64.1)	-	-
Runny or blocked nose	393 (59.8)	-	-
Sore throat	235 (35.8)	-	-
Loss of smell	27 (4.1)	-	-
Loss of appetite	50 (7.6)	-	-
Diarrhea	50 (7.6)	-	-
Headache	205 (31.2)	-	-
Vomiting	32 (4.9)	-	-
Others *	145 (22.1)	-	-
There is currently no effective cure for COVID-19, but early symptomatic and supportive treatment can help most patients recover from the infection	638 (97.1)	10 (1.5)	9 (1.4)
Not all persons with COVID-19 will develop severe disease. Only those who are elderly and have chronic illnesses are more likely to develop severe disease	545 (83)	102 (15.5)	10 (1.5)
Persons with COVID-19 cannot transmit the virus to others if they do not have a fever (False)	43 (6.5)	604 (91.9)	10 (1.5)
The COVID-19 virus spreads via respiratory droplets of infected individuals	631 (96)	15 (2.3)	11 (1.7)
Wearing general facemasks can prevent one from acquiring infection by the COVID-19 virus	631(96)	21(3.2)	5 (0.8)
It is not necessary for children and young adults to take measures to prevent infection by the novel coronavirus (False)	47 (7.2)	601 (91.5)	9 (1.4)
To prevent infection by COVID-19, individuals should avoid going to crowded places and avoid using public transport	608 (92.5)	39 (5.9)	10 (1.5)
There is currently no effective vaccine for COVID-19	605 (92.1)	21 (3.2)	31 (4.7)

N = Number; % = percentage; * Chest pain, sneezing, red eyes, abdominal pain, hoarse voice.

**Table 3:** Descriptive summary of COVID-19 attitudes among HCWs in Uganda.

Attitude Questions (N = 657)	Agree	Disagree	Not Sure
	N (%)	N (%)	N (%)
COVID-19 will be successfully controlled	535 (81.4)	30 (4.6)	92 (14)
Uganda will win the battle against COVID-19	522 (79.5)	40 (6.1)	95 (14.5)
You would feel confident participating in the management of a patient who has signs and symptoms of COVID-19 (*n* = 420) ^§^	341 (81.2)	52 (12.4)	27 (6.4)
You would trust that wearing a well-fitting face mask is effective in preventing COVID-19	609 (92.7)	28 (4.3)	20 (3)
A safe and effective vaccine is the best hope for eliminating COVID-19	575 (87.5)	29 (4.4)	53 (8.1)
Africans should participate in studies evaluating COVID-19 vaccines	633 (96.4)	11 (1.7)	13 (2)
Healthcare workers should be given priority for vaccination if a safe and effective COVID-19 vaccine is found	628 (95.6)	13 (2)	16 (2.4)

N = Number; % = percentage; ^§^ Excludes 232 participants whose work did not involve direct contact with patients and 5 participants with missing responses.

**Table 4:** Descriptive summary of COVID-19 practices among HCWs in Uganda.

Practice Questions (N = 657)	Responses			
	Twice or More Every Day	Once A Day	Not Every Day	Never
	N (%)	N (%)	N (%)	N (%)
In the last two weeks, how many times have you joined gatherings with people other than your household members?	120 (18.3)	75 (11.4)	190 (28.9)	272 (41.4)
In the last two weeks, how many times have you washed your hands with soap and water or cleaned them using hand sanitizer?	657 (100)	-	-	-
	Never	Always	Occasionally	
In the last two weeks, how often have you refrained from shaking hands	29 (4.4)	563 (85.7)	65 (9.9)	
In the last two weeks, how often have you worn a mask when at work?	3 (0.5)	595 (90.5)	59 (9.0)	
In the last two weeks, how often have you washed your hands with soap and water or cleaned them with hand sanitizer before and after handling each patient? (*n* = 412) ^ƪ^	1 (0.2)	395 (95.9)	16 (3.9)	
In the last two weeks, how often have you avoided patients with signs and symptoms suggestive of COVID-19 (*n* = 408) ^β^	235 (57.6)	122 (29.9)	51 (12.5)	

N = Number; % = percentage; ^ƪ^ Excludes 232 participants whose work did not involve direct contact with patients and 13 participants with missing responses; ^β^ Excludes 232 participants whose work did not involve direct contact with patients and 17 participants with missing responses.

**Table 5:** Factors associated with knowledge, attitude and practice toward COVID-19 among healthcare workers in Uganda.

	Sufficient Knowledge		Positive Attitude		Good Practices	
Characteristic	N (Row %)	aRR (95% CI)	N (Row %)	aRR (95% CI)	N (Row %)	aRR (95% CI)
**Age group**						
18–25 years	132 (82.5)		134 (83.8)		60 (37.5)	
26–35 years	248 (85.5)	1.03 (0.95–1.12)	229 (79.0)	0.95 (0.87–1.05)	107 (36.9)	1.13 (0.88–1.45)
>35 years	172 (83.1)	1.05 (0.95–1.16)	152 (73.4)	0.88 (0.79–0.98) *	76 (36.7)	1.25 (0.95–1.64)
**Sex**						
Male	210 (85.4)		197 (80.1)		80 (32.5)	
Female	345 (83.9)	0.96 (0.89–1.02)	318 (77.4)	0.95 (0.87–1.03)	163 (39.7)	1.07 (0.86–1.33)
**Occupation of the respondents**						
Non-clinical HCW	174 (75.0)		174 (75.0)		53 (22.8)	
Clinical HCW	381 (89.7)	1.12 (1.02–1.23) *	341 (80.2)	1.11 (0.98–1.25)	190 (44.7)	1.91 (1.41–2.59) *
**Education level**						
Ordinary secondary school and below	95 (72.5)		106 (80.9)		39 (29.8)	
Advanced secondary school/other higher-level	338 (89.0)	1.12 (0.99–1.26)	301 (79.2)	0.93 (0.82–1.06)	162 (42.6)	0.91 (0.66–1.25)
University	122 (83.6)	1.06 (0.92–1.21)	108 (74.0)	0.91 (0.79–1.06)	42 (28.8)	0.73 (0.49–1.07)
**Previously volunteered to participate in health research**						
No	428 (82.0)		414 (79.3)		192 (36.8)	
Yes	127 (94.1)	1.10 (1.04–1.17) *	101 (74.8)	0.93 (0.84–1.04)	51 (37.8)	0.95 (0.74–1.20)
**Use social media as source of information**						
No	153 (78.1)		156 (79.6)		57 (29.1)	
Yes	402 (87.2)	1.05 (0.97–1.14)	359 (77.9)	0.96 (0.86–1.06)	186 (40.4)	1.24 (0.96–1.61)

Only variables that attained a significance level of 0.15 using the Likelihood Ratio Test at unadjusted analysis were included in this table; N = Number; % = percentage; aRR = adjusted relative ratio; CI = confidence interval; * *p*-value < 0.05.

## Data Availability

Data supporting the conclusions of this article can be retrieved following the data sharing policy at the MRC/UVRI & LSHTM Uganda Research Unit. This policy can be assessed through the following link: https://www.mrcuganda.org/publications/data-sharing-policy. (accessed on 17 June 2021).
